# Application of Bayesian evidence synthesis to modelling the effect of ketogenic therapy on survival of high grade glioma patients

**DOI:** 10.1186/s12976-018-0084-y

**Published:** 2018-08-20

**Authors:** Rainer J. Klement, Prasanta S. Bandyopadhyay, Colin E. Champ, Harald Walach

**Affiliations:** 1 Department of Radiotherapy and Radiation Oncology, Leopoldina Hospital Schweinfurt, Robert-Koch-Straße 10, 97422 Schweinfurt, Germany; 2 Department of History & Philosophy, Montana State University, Bozeman, MT USA; 3 Department of Radiation Oncology, University of Pittsburgh Medical Center, Pittsburgh, PA USA; 4 Department of Pediatric Gastroenterology, Medical University Poznan, Poznan, Poland; 5 Department of Psychology, University Witten-Herdecke, Witten, Germany

**Keywords:** Bayesian evidence synthesis, Calorie restriction, Evidence based medicine, High grade glioma, Ketogenic diet, Philosophy of medicine, Philosophy of science

## Abstract

**Background:**

Ketogenic therapy in the form of ketogenic diets or calorie restriction has been proposed as a metabolic treatment of high grade glioma (HGG) brain tumors based on mechanistic reasoning obtained mainly from animal experiments. Given the paucity of clinical studies of this relatively new approach, our goal is to extrapolate evidence from the greater number of animal studies and synthesize it with the available human data in order to estimate the expected effects of ketogenic therapy on survival in HGG patients. At the same time we are using this analysis as an example for demonstrating how Bayesianism can be applied in the spirit of a circular view of evidence.

**Results:**

A Bayesian hierarchical model was developed. Data from three human cohort studies and 17 animal experiments were included to estimate the effects of four ketogenic interventions (calorie restriction/ketogenic diets as monotherapy/combination therapy) on the restricted mean survival time ratio in humans using various assumptions for the relationships between humans, rats and mice. The impact of different biological assumptions about the relevance of animal data for humans as well as external information based on mechanistic reasoning or case studies was evaluated by specifying appropriate priors. We provide statistical and philosophical arguments for why our approach is an improvement over existing (frequentist) methods for evidence synthesis as it is able to utilize evidence from a variety of sources. Depending on the prior assumptions, a 30–70% restricted mean survival time prolongation in HGG patients was predicted by the models. The highest probability of a benefit (> 90%) for all four ketogenic interventions was obtained when adopting an enthusiastic prior based on previous case reports together with assuming synergism between ketogenic therapies with other forms of treatment. Combinations with other treatments were generally found more effective than ketogenic monotherapy.

**Conclusions:**

Combining evidence from both human and animal studies is statistically possible using a Bayesian approach. We found an overall survival-prolonging effect of ketogenic therapy in HGG patients. Our approach is best compatible with a circular instead of hierarchical view of evidence and easy to update once more data become available.

**Electronic supplementary material:**

The online version of this article (10.1186/s12976-018-0084-y) contains supplementary material, which is available to authorized users.

## Background

High grade glioma (HGG) is the most common brain tumor among adults, and is comprised of astrocytoma, oligodendroglioma and glioblastoma multiforme (GBM). GBM has the worst prognosis, with median survival rates of approximately 15–20 months when treated with the standard approach of surgical resection followed by photon radiotherapy to 60 Gy with simultaneous temozolomide administration [1, 2]. Given these poor outcomes, there is an urgent need for alternative and complementary treatment approaches. One such approach that has gained proponents in recent years is ketogenic therapy which targets the metabolic differences between malignant tumor and normal brain cells by shifting the cells’ substrate availability from glucose towards fatty acids and ketone bodies [3–7]. This concept is supposed to induce energetic stress in tumor cells, which in case of malignant brain tumors have frequently been shown to be deficient in key ketolytic enzymes, in particular 3-hydroxybutyrate dehydrogenase 1 (BDH1) and 3-oxoacid-CoA transferase 1 (OXCT1) [8–10], although counterexamples exist [11]. This chronic energy stress should make tumor cells more vulnerable to acute stresses such as those induced by oxidative therapies (chemo-, radio-, hyperbaric oxygen therapy) [12, 13].

Ketogenic metabolic therapy, or more shortly ketogenic therapy, is an umbrella term comprising ketogenic diets (KDs), calorie restriction (CR), the extreme form of CR which is short-term fasting, and application of exogenous ketone bodies [7, 13, 14]. All these modalities increase circulating ketone body levels and are able to lower glucose and insulin levels. Combined and individually, these metabolic changes are predicted to alter tumor signaling networks, exerting putative actions against tumor growth not only in brain cancer [4, 5] but also other solid malignancies such as head and neck cancer [15], breast cancer [16] or advanced thyroid cancer [17]. However, while these reviews optimistically emphasized the evidence available from preclinical studies, case studies, small clinical pilot studies and basic mechanistic reasoning^1^ there currently appears to be no “real” evidence for this approach within the classical evidence-based medicine (EBM) framework which in its standard form demands (systematic reviews of) high-quality, randomized clinical trials for “*really telling scientific evidence”* [18]. Although some authors have recognized that in many clinical situations also other types of evidence are sufficient or need to be considered as well [19, 20], the common consensus is still, though not uncontested [18, 21], that only randomized controlled trials (RCTs) and their meta-analytical summaries are “real” evidence.

This situation is problematic, because patients frequently value ketogenic therapy as a putatively beneficial and safe treatment that returns agency and self-care and thereby empowerment to them. For example, a recent survey among HGG patients found that almost three quarters of them (73%) would be willing to test a KD for 3 months [22]. Nevertheless EBM enthusiasts discourage its use because scientific evidence is lacking [23]. This is despite the fact that EBM in principle acknowledges that other types of (what is called “next best”) evidence exist that should be considered in case that RCTs are not available. Furthermore, other types of evidence are supposed to be incorporated into an overall judgment, although it is not made explicit how this should be done in practice [18].

Four specific problems exist which make finding a solution for the question how to best amalgamate evidence from different sources challenging. First, as pointed out by Thompson and Upshur, “*the vast majority of clinical researchers, regulatory agencies and clinical practitioners either reject, ignore or are oblivious to … criticisms [of the superiority of RCTs for evidence generation]. We attribute this to entrenchment*” ([24], page 83). Second, the epistemology of EBM and RCTs is firmly grounded in a frequentist interpretation of probability [24, 25]. This makes an amalgamation of different sources of evidence or different outcome measures into a meta-analysis difficult due to relative inflexibility of the frequentist framework [26, 27].^2^ Third, although EBM claims to be based on evidence, it lacks a sound theoretical concept of evidence itself [28], in particular concerning its methods of statistical inference [25]. Assuming that the theory of evidence in medicine should conform to that in other sciences, the hierarchical view of evidence that prevails in EBM seems to violate this assumption [18]. For instance, in physics evidence for a hypothesis is accepted, if a certain amount of independent observers, irrespective of the particular methodology used, agree on a phenomenon predicted by the hypothesis, and the probability of observing the data or more extreme values, given that the hypothesis is false, is low, usually lying more than a few standard deviations away from the mean. In other words, the observed data would be much more likely under the hypothesis than they would be under its rival hypotheses. In medicine, there is no comparable stringency [29], and the evidence hierarchy seems to us to simulate stringency built on shaky theoretical ground. Fourth, the frequentist approach to any statistical problem makes a highly improbable and unnatural assumption, namely that a hypothesis is either true or false [30], corresponding to a probability of either 1 or 0, irrespective of the theoretical grounding of a particular research or the mechanistic knowledge or the clinical experience. Thus this approach excludes an important human property from decision making: the intelligent summarizing of empirical findings against the background of known or likely facts and theories.

A promising concept that allows a solution to these four problems has been proposed by Walach et al. [31] in form of a circular view of evidence, in which different study designs would contribute different and partly complementary “pieces of evidence” for the generation of an overall epistemic picture. While the hierarchical approach assumes that evidence generated by internally valid methods, such as RCTs, is preferable over evidence generated by externally valid methods, such as observational or epidemiological studies, the circular view acknowledges that each method has strengths and weaknesses that can only be balanced by taking all the evidence together.

This approach overcomes the entrenchment which prevails among the medical community and is compatible with a basic scientific concept of evidence. We here adopt the concept of evidence as a measure of distinction between two competing hypotheses [32], something that could in principle be established in a variety of study types by considering the study-specific background information or context which would provide clues for deciding whether one of two hypotheses is more likely given the data and our background knowledge. A circular view of evidence is also compatible with a more general approach to evidence synthesis such as the matrix-analytical approach proposed by Walach and Loef [33] or flexible Bayesian meta-analytical methods [27, 34–37]. The matrix analytical approach would tabulate all the available data according to design and questions answered, potentially including the methodological rigor of various studies. The Bayesian method takes into account prior knowledge and background theory. Combining these two approaches might yield a powerful, more flexible and adequate method of data synthesis.

We here aim to demonstrate such a flexible Bayesian approach to evidence synthesis using the available data on ketogenic therapy against HGG as an example. Specifically, given the paucity of clinical studies of this relatively new approach, our goal is to utilize information from observational studies and the large amount of preclinical animal studies, incorporating various assumptions about the relevance of these data for humans into our model. We consider our approach important as an evidence summary reflecting the current state of research that could aid decision making of clinicians and patients alike, and as an evidence base that can be updated anytime once more studies, preferably in humans, become available. At the same time this somewhat controversial area serves as a testing ground for the purported flexibility of a combined approach, using Bayesian reasoning and cross-fertilizing data from various methodological approaches.

## Methods

### Bayesian evidence synthesis

Our approach is based on methodology developed by DuMouchel and Harris [34], DuMouchel and Groër [38] and Jones et al. [39]. These authors integrated findings from animal and human studies on dose-response relationships between certain toxic exposures and the risk of complications, quantified by the slope of the dose-response curves. Underlying these models is the assumption that all experiments are “*related through some unifying biological hypothesis*” and that “*the results of each experiment are summarized by a single number, such as the slope of the dose-response relation*.” [34]. We here adopt the restricted mean survival time (RMST) ratio (henceforth simply denoted as MR) between the intervention and control group in each study as this single outcome measure we seek to combine. While the mean survival time for a random time-to-event variable *T* is given as $\mu ={\int}_0^{\infty }S(t) dt$, the RMST measures the area under the survival curve up to a specified time point: $\mathrm{RMST}\left({t}^{\ast}\right)=\mathrm{E}\left[\min \left(T,{t}^{\ast}\right)\right]={\int}_0^{t^{\ast }}S(t) dt$. The RMST is therefore a measure of average survival up to the specified follow-up time. For the specified time point, we adopt the last recorded time point in each study group, so that in cases in which all subjects experience the event of interest (like in most animal studies), the RMST is equal to the mean survival time. Spruance et al. have argued that in such cases, time parameters such as mean survival times provide a more useful description of group survival statistics than the hazard ratio [40]. Furthermore, an advantage of the RMST is that it is valid under any distribution of the time to event in the treatment groups, of which proportional hazards models are only a (small) sub-class; it is therefore an alternative to the hazard ratio whenever the proportional hazards assumption is doubtful [41, 42].

The MRs between intervention and control groups were converted to the logarithmic scale such that ln(MR) > 0 indicates longer survival in the treatment group. The unifying biological hypothesis consists in assuming an “equal relative potency” of the interventions, meaning that the ratio between the MRs of any two interventions is preserved across species. For example, if combining CR with radiotherapy in a given strain of mice prolongs survival by a factor of 2 compared to radiotherapy only, equal relative potency implies that the same factor should apply to other mouse strains or humans.

### Data

All the data were retrieved from studies included in previous systematic reviews on KDs and cancer, specifically the meta-analysis of mouse studies by Klement et al. [43], the review on the role of ketogenic therapy in HGG by Winter et al. [7] and the realist review by Klement [44]. Searches in PubMed using the search terms “ketogenic diet”, “calorie restriction”, “caloric restriction”, “glioma”, “glioblastoma” and “astrocytoma” up to December 2017 revealed no additional studies. Individual survival times were retrieved for most studies from the Kaplan-Meier graphs using the software Digitizeit version 2.3.2, while for some studies authors delivered the survival data upon request. Details of the studies are given in Table 1. The studies could be categorized into four ketogenic intervention groups: KD as monotherapy, KD combined with other treatment (KD+), calorie restriction as monotherapy and calorie restriction combined with other treatment (CR+). A total of 17 animal experiments from 10 papers [9, 45–53] allowed retrieval of individual survival times from which RMSTs with standard errors of ketogenic and control animals were calculated.^3^ For animal species, we differentiated different genera and strains, but not sub-strains or gender so that the total number of species considered in the model was five (humans, athymic mice, C57BL/6 mice, SCID mice, Fisher rats). As in our previous meta-analysis [43], we required animal studies to adopt a pre-specified survival outcome such as growth to a certain tumor volume but no pre-specified study termination time point. If multiple studies were available for a given species and intervention, we pooled the individual study outcomes together using Bayesian random effects meta-analysis with three different priors for the between-study variance (uniform, half-normal and DuMouchel’s prior) as described in Klement et al. [43]. This was done as the model required exactly one datum per intervention and species.

**Table 1:** Study data used for the evidence synthesis

Year	First author	Species	Tumor	Treatment	N_T_ + N_C_	RMST_T_	RMST_C_	MR	Data source
2014	Han	Humans	GBM	CR+	11 + 23	41.36 ± 4.02	23.70 ± 2.85	1.75 ± 0.27	Figure
2015	Rieger	Humans	GBM	KD	8 + 5	7.25 ± 1.07	5.16 ± 1.29	1.41 ± 0.41	Figure
2018	Santos	Humans	GBM	KD+	17 + 15	10.56 ± 0.94	8.38 ± 1.49	1.26 ± 0.25	Author
2007	Zhou	C57BL/6J mice	CT-2A astrocytoma (syn.)	KD	9 + 7	19.78 ± 0.84	17.71 ± 0.94	1.12 ± 0.08	Author
2007	Zhou	C57BL/6J mice	CT-2A astrocytoma (syn.)	CR	11 + 7	31.82 ± 2.26	17.71 ± 0.94	1.80 ± 0.16	Author
2007	Zhou	BALBc/J SCID mice	U87 glioma (xen.)	KD	7 + 11	19.71 ± 0.78	21.27 ± 1.84	0.93 ± 0.09	Author
2007	Zhou	BALBc/J SCID mice	U87 glioma (xen.)	CR	14 + 11	30.36 ± 1.40	21.27 ± 1.84	1.43 ± 0.14	Author
2008	Marsh	C57BL/6J mice	CT-2A astrocytoma (syn.)	CR	8 + 7	30.50 ± 4.09	18.86 ± 1.20	1.62 ± 0.24	Figure
2010	Stafford	C57BL/6J albino mice	GL261 glioma (syn.)	KD	5 + 5	24.00 ± 0.94	19.00 ± 0.63	1.26 ± 0.06	Author
2011	Maurer	Athymic mice	LNT-229 glioma (xen.)	KD	12 + 12	87.83 ± 4.59	96.90 ± 3.57	0.91 ± 0.06	Author
2012	Abdelwahab	C57BL/6J albino mice	GL261 glioma (syn.)	KD	20 + 19	33.90 ± 5.12	23.32 ± 1.08	1.45 ± 0.23	Author
2012	Abdelwahab	C57BL/6J albino mice	GL261 glioma (syn.)	KD+	11 + 11	97.36 ± 4.93	54.73 ± 9.81	1.78 ± 0.33	Figure
2013	Jiang	Athymic mice	U87 glioma (xen.)	CR	7 + 7	26.71 ± 1.55	18.71 ± 0.85	1.43 ± 0.11	Figure
2014	Rieger	Athymic mice	U87 glioma (xen.)	KD	8 + 8	35.62 ± 0.64	33.88 ± 1.53	1.05 ± 0.05	Author
2014	Rieger	Athymic mice	U87 glioma (xen.)	KD+	8 + 8	57.50 ± 2.28	50.25 ± 2.24	1.14 ± 0.07	Figure
2015	Martuscello	NOD/SCID mice	L0 glioma (xen.)	KD	10 + 11	48.50 ± 5.63	14.55 ± 1.57	3.33 ± 0.53	Figure
2015	Martuscello	NOD/SCID mice	L2 glioma (xen.)	KD	5 + 6	55.20 ± 8.15	25.5 ± 2.38	2.17 ± 0.38	Figure
2016	De Feyter	Fisher rats	9 L glioma (xen.)	KD	10 + 9	34.70 ± 1.63	33.56 ± 1.33	1.07 ± 0.07	Author
2016	De Feyter	Fisher rats	RG2 glioma (xen.)	KD	10 + 11	26.30 ± 1.11	27.36 ± 0.71	0.96 ± 0.05	Author
2016	Lussier	C57BL/6J albino mice	GL261 glioma (syn.)	KD	12 + 11	42.17 ± 2.52	33.09 ± 1.45	1.27 ± 0.09	Figure

Subscript “T” denotes treatment group, “C” control group. *CR/KD* Calorie restriction/ Ketogenic diet as monotherapy, *CR+/KD+* Calorie restriction/ ketogenic diet combined with another therapy, *syn.* syngeneic transplant, *xen.* xenogeneic transplant

**Fig. 1:**
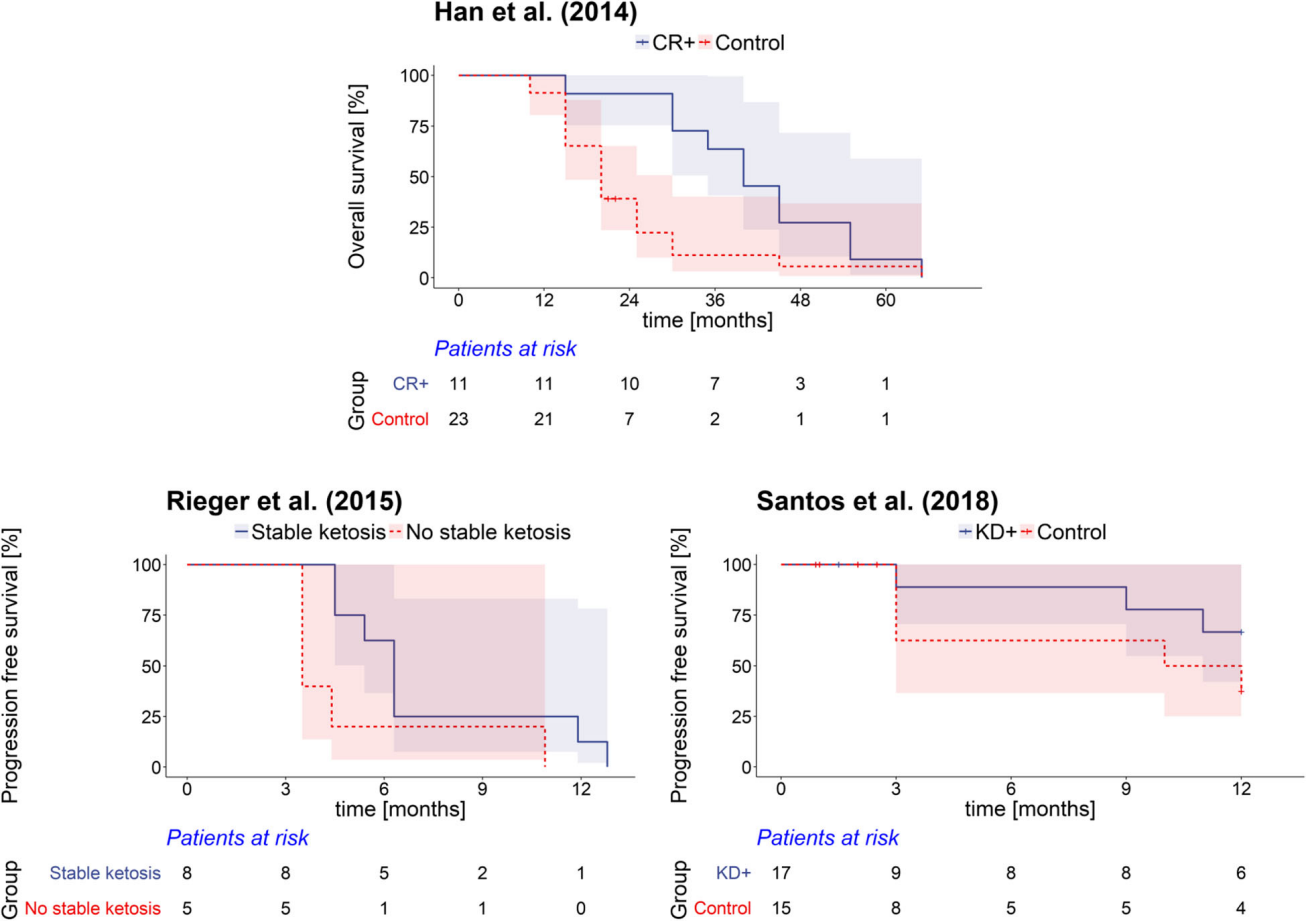
Kaplan-Meier plots of the three human studies used in this analysis. The 95% confidence intervals are also shown as shaded areas. The curves from the studies of Han et al. and Rieger et al. are based on data retrieved from the Kaplan-Meier plots in the original publications, while that corresponding to the study of Santos et al. is based on data provided by Dr. Juliana Guimarães Santos

Only three human studies provided survival outcomes that allowed computing a MR between a “ketogenic” and a “non-ketogenic” group: Han et al. [54] combined CR with hyperbaric oxygen, psychotherapy and standard of care (radio-chemotherapy) as a treatment of newly diagnosed GBM patients and compared overall survival against a control group that received standard of care only. The ERGO study by Rieger et al. [50] prescribed a KD as a monotherapy for patients with recurrent GBM who already had received standard of care but had no planned control group. However, since a number of patients did not reach stable ketosis, we compared their outcome (progression-free survival) against that of the patients who were in stable ketosis based on Figure 5A in Rieger et al. [50]. Since there was no genuine standard diet control group, the effect of omitting this study on the MR posterior estimates was assessed in a sensitivity analysis. Finally, Santos et al. conducted a clinical trial of intranasal application of perillyl alcohol combined with a KD against only perillyl alcohol delivery in post-surgical recurrent GBM patients with no further treatment options. The outcome of interest was progression-free survival, while patients that died within the first three months (the prescribed KD duration) or did not conform to the KD were treated as censored. Individual survival times and information was kindly provided by Dr. Juliana Guimarães Santos. Figure 1 exemplarily shows the Kaplan-Meier survival curves of the three human studies; those of the animal studies can be looked up in the original publications. The individual study data that our analysis is based on are given in Table 2.

**Table 2:** Estimates of ln(MR) ± standard deviation from individual studies according to species and intervention

Species/Intervention	KD	KD+	CR	CR+
Humans	0.3436 ± 0.2842	0.2311 ± 0.1972	NA	0.5596 ± 0.1532
Athymic mice	−0.0186 ± 0.3529	0.1345 ± 0.0305	0.3563 ± 0.0376	NA
C57BL mice	0.2261 ± 0.0956	0.576 ± 0.0961	0.5415 ± 0.3219	NA
SCID mice	0.6325 ± 0.5048	NA	0.3556 ± 0.0501	NA
Fisher rats	0.0117 ± 0.3228	NA	NA	NA

*CR/KD* Calorie restriction/Ketogenic diet as monotherapy, *CR+/KD+* Calorie restriction/ketogenic diet combined with another therapy

### Philosophical aspects

We assume a moderate ontological pluralism in which each study type exists “in its own universe”, yet is able to generate effect sizes that constitute evidence for or against the hypothesis that ketogenic therapy increases HGG survival and can be amalgamated into an overall estimate, consistent with a more circular view of evidence [31]. The standpoint of moderate pluralism implies that different study types can have different access routes to the underlying reality, thus offering multiple perspectives on what counts as surviving/not surviving depending on the outcome of a specific experiment. In this sense, allowing for an ontological pluralism justifies the summation and combination of each study by a single number, in our case the MR.

Furthermore, the Bayesian framework allows us to use the case studies which do not provide MRs, but still could provide evidence for efficacy within their own specific context [44], to formulate prior expectations about the MRs observed within the cohort studies. Analogously, mechanistic reasoning, while usually excluded from the evidence hierarchy of EBM [19], could be accounted for using appropriate priors. In this way, the Bayesian approach is flexible enough to account for a circular instead of hierarchical approach to meta-analysis. As Jones et al. stated: *“Other information about species differences, physical and metabolic characteristics of the exposures, disease mechanisms, and so on may be represented in the prior distributions of hyperparameters in the Bayesian model”* [39].

In fact, we argue that Bayesianism is the only statistical school able to provide a unifying philosophical account of scientific reasoning through its ability to address three fundamental questions of scientific inference [32, 55]: (i) Given the data, what should we believe and to what degree (the belief question)? (ii) Given the data, what is the evidence for our hypothesis that ketogenic therapy has a survival-prolonging effect in HGG patients, equivalent to ln(MR) > 0 (the evidence question)? (iii) What should we do (the decision question)? See Additional file 1: Appendix A for more information and our justification for the Bayesian approach.

### The model

For our Bayesian model, we adopt the notation given by Jones et al. [39]. We assume a normal likelihood for the ln(MR) in the *i*th row (species) and *j*th column (intervention):

$$ {y}_{ij}\sim N\left({\theta}_{ij},{c}_{ij}^2\right) $$

where

$$ {\theta}_{ij}\sim N\left({\alpha}_i+{\gamma}_j,{\sigma}^2\right), $$

$$ {\alpha}_i\sim N\left({\mu}_{\alpha_i},{\sigma}_{\alpha_i}^2\right), $$

$$ {\gamma}_j\sim N\left({\mu}_{\gamma_j},{\sigma}_{\gamma_j}^2\right). $$

Thus, *θ*_*ij*_ is the true ln(MR) and ${c}_{ij}^2$ the variance of *y*_*ij*_ for species *i* and intervention *j*. The variables *α*_*i*_ and *γ*_*j*_ represent the species and intervention effects, respectively, and *σ*^2^ measures how well the assumption of equal relative potency of interventions across species fits the data [39].

### Prior distributions

As noted above, prior distributions on the hyperparameters *α*_*i*_, *γ*_*j*_ and *σ*^2^ allow us to incorporate prior knowledge on disease mechanisms, metabolic effects and any information or belief about inter-species relationships from external sources. We can also model the prior beliefs of ketogenic therapy proponents as against skeptics, and assess the different impact on the results in sensitivity analyses. Accordingly, we specify different prior distributions for the relationship between the species effects, *α*_*i*_, and the intervention effects, *γ*_*j*_. The relationships of species and intervention effects are specified through covariance matrices Ralpha and Rgamma, respectively [39].

### Prior for *σ*

The parameter *σ* gauges the accuracy of the equal relative potency assumption by measuring the deviations of the *α*_*i*_ + *γ*_*j*_ from the true effects *θ*_*ij*_. Thus, the assumption that the ratio between the MRs of two interventions is preserved across species is only accurate to within a deviation of exp(±*σ*) with 68% probability [34]. We use a prior distribution *σ*~*N* (0.5,0.01) as in Jones et al. [39], implying that with 68% probability, the constant relative potency assumption would be accurate to within a factor ranging from exp(0.4) ≈ 1.5 to exp(0.6) ≈ 1.8 with a factor of exp(0.5) ≈ 1.65 being most likely.

### Skeptical priors

Skeptical priors assume that none of the species and interventions yields information relevant to the others. They have a diagonal structure. We use two skeptical priors: SP1 specifies rather vague priors for both species and intervention effects, *α*_*i*_~*N*(0, 10) and *γ*_*j*_~*N*(0, 10), following DuMouchel and Groër [38] and Jones et al. [39] and implying a variance of the true effects of 100 + *σ*^2^. SP2 specifies weakly informed priors *α*_*i*_~*N*(0, 1) and *γ*_*j*_~*N*(0, 1), implying a variance of the true study effects of 1 + *σ*^2^.

### Fundamentalist skeptical priors

“Fundamentalist skeptics”^4^ would argue that ketogenic therapy could accelerate tumor growth, resulting in decreased survival times. We model such beliefs by the priors FSP: *α*_*i*_~*N*(−0.35,1),*γ*_*j*_~*N*(−0.35,1) and a diagonal structure of the covariance matrices assuming no information of one species or intervention is relevant to others as in SP1 or SP2 above. Together, these priors correspond to the expectation of the survival time being decreased by a factor of ≈2 in a group treated with ketogenic therapy.

### Relational priors

With a relational prior on Rgamma, we take into account the similar metabolic effects of CR and KDs [56] by assigning a correlation of 0.9 between KD and CR as well as between KD+ and CR+:

$$ \mathrm{Rgamma}=\left(\begin{array}{cccc}10& 0& 9& 0\\ {}0& 10& 0& 9\\ {}9& 0& 10& 0\\ {}0& 9& 0& 10\end{array}\right), $$

In the relational prior RP1, we additionally assume strong correlations between the three mouse strains which we express by off-diagonal elements in the 5 × 5 matrix Ralpha using a correlation coefficient of 0.9995 as in Jones et al. [39]:

$$ \mathrm{Ralpha}=\left(\begin{array}{l}10\kern1.75em 0\kern2.25em 0\kern2.25em 0\kern1.75em 0\\ {}\ 0\kern1.5em 10\kern1em 9.995\kern0.75em 9.995\kern1em 0\\ {}\ 0\kern0.75em 9.995\kern1.25em 10\kern1.25em 9.995\kern1em 0\\ {}\ 0\kern0.75em 9.995\kern0.5em 9.995\kern1.25em 10\kern1.75em 0\\ {}\ 0\kern1.75em 0\kern2.5em 0\kern2.25em 0\kern1.5em 10\end{array}\right). $$

We also specify a second relational prior RP2 which additionally assumes a correlation between mice and rats of 0.9, corresponding to a standard deviation of 1.41 for the difference *α*_mouse_ − *α*_rat_. Finally, a third relational prior RP3 assumes some biological relevance between the animal models and humans, although we consider this weak based on the exaggerated metabolic effects compared to humans when rodents are put on calorically restricted diets [57], as well as differences in blood glucose regulation [58]; we thus apply (arbitrarily) a correlation of 0.3 and check the impact of this choice in sensitivity analyses.

### Mechanistic prior for Rgamma

Mechanistic reasoning suggests that ketogenic therapy is able to boost the effectiveness of other anti-cancer treatments [12–14, 59] which we model through the relations *γ*_2_ = *γ*_1_ + *η* and *γ*_4_ = *γ*_3_ + *η* with *η*~*N*(0.3,0.3), and a weakly informative prior on the *γ*_*j*_'s according to *γ*_*j*_~*N*(0, 1). This prior expresses the assumption that the average MR for KD+/CR+ is exp(0.3) ≈ 1.35, i.e. a 35% increased survival time compared to no additional ketogenic treatment. In this basic mechanistic prior (denoted MP1) Rgamma is therefore

$$ \mathrm{Rgamma}=\left(\begin{array}{l}1\kern1.25em 0\kern1.25em 0\kern1.25em 0\\ {}0\kern0.5em 1.3\kern1em 0\kern1.25em 0\\ {}0\kern1em 0\kern1.25em 1\kern1.25em 0\\ {}0\kern1em 0\kern1.25em 0\kern0.75em 1.3\ \end{array}\right). $$

We can also combine the mechanistic prior with the correlations assumed for the treatments as in the relational priors, so that in MP2:

$$ \mathrm{Rgamma}=\left(\begin{array}{l}\kern0.5em 1\kern1.75em 0\kern2em 0.99\kern2em 0\\ {}\kern0.5em 0\kern1.5em 1.3\kern2em 0\kern1.5em 1.287\\ {}0.99\kern1em 0\kern2.5em 1\kern2.75em 0\\ {}\kern0.5em 0\kern1.25em 1.287\kern1.25em 0\kern2.25em 1.3\ \end{array}\right) $$

Both MP1 and MP2 assume

*μ*_*γ*1_ = *μ*_*γ*3_ = 0 and *μ*_*γ*2_ = *μ*_*γ*4_ = 0.3.

### Enthusiastic prior for Ralpha

Klement has compiled cohort and case studies on KDs in cancer patients and asked whether each of them provides evidence for an anti-tumor hypothesis against the hypothesis of no effect [44]. There are a total of seven case studies in his compilation related to treatment of patients with astrocytoma and/or GBM. Out of these, three were judged as providing evidence for the anti-tumor hypothesis [60–62], three were inconclusive [63–65] and one clearly provided no evidence [11]. No study provided evidence for any pro-tumor effects. Thus, based on these published case reports, and consciously neglecting any publication bias associated with these case reports, we formulate an optimistic prior for an anti-tumor effect of KDs at least in some patients which we express as the expectation that ketogenic therapy combined with other treatments could prolong survival by 40% with 3/7 ≈ 42.9% confidence that the MR will lie between 1.1 and 2, favoring KDs. On the log-scale, adopting a normal distribution this corresponds to a standard deviation of [ln(2) − ln(1.1)]/(2 × 0.79) = 0.378, with mean at ln(1.5) = 0.336. For mice, we further adopt the findings of the meta-analysis of Klement et al. [43] which revealed a prolongation of mean survival times in mice bearing brain tumors that were treated with KD monotherapy which can be translated to a normal distribution on the log-scale with mean value ln(1.124) = 0.117 and standard deviation [ln(1.136) − ln(0.962)]/(2 × 1.96) = 0.080 (note that the MR in Klement et al. [43] was defined as the ratio between mean survival times in the control versus the ketogenic group, which is the reciprocal of the MR adopted here). Thus, we apply *α*_1_~*N*(0.336, 0.378^2^) and *α*_2,3,4_~*N*(0.117, 0.08^2^) together with a weakly informative prior *α*_5_~*N*(0, 1) for the rats, so that the enthusiastic prior (EP) is

$$ \mathrm{Ralpha}=\left(\begin{array}{l}\ 0{.378}^2\kern2em 0\kern3.25em 0\kern3em 0\kern2.5em 0\\ {}\kern1em 0\kern2.5em 0{.08}^2\kern1.5em 0.0063\kern0.75em 0.0063\kern1.25em 0\\ {}\kern1em 0\kern2em 0.0063\kern1.75em 0{.08}^2\kern1em 0.0063\kern1.25em 0\\ {}\kern1em 0\kern2em 0.0063\kern1.25em 0.0063\kern1.5em 0{.08}^2\kern1.25em 0\\ {}\kern1em 0\kern3.25em 0\kern3.25em 0\kern3em 0\kern2.75em 1\ \end{array}\right). $$

### Model inference

Models were run in WinBUGS version 1.4. Each time, the first 100,000 iterations were considered as burn-in samples and discarded, and the next 200,000 iterations were sampled for inference using a thinning of 20 in order to reduce auto-correlation. From the resulting 10,000 samples, medians were taken as point estimates and 95% credible intervals (CrIs) derived. A sample code for the model with the enthusiastic prior is given in Additional file 1: Appendix B.

### Plausibility of prior assumptions

The plausibility of the various priors was checked using the Deviance Information Criterion (DIC) [66, 67]. DIC is defined as the sum of the posterior mean deviance $\overline{D}$ (a measure of model adequacy) and the effective number of parameters *p*_*D*_ (a measure of model complexity). An information-theoretical perspective on DIC has been given by van der Linde [68]. She showed that for sampling distributions belonging to the exponential family, the complexity term *p*_*D*_ in DIC can be interpreted as an estimate of the symmetrized mutual information between future observations and posterior parameter estimates. Compared to Bayes factors, DIC is more appropriate to compare hierarchical models; it measures what the data tell us about the predictive accuracy rather than the truth of a model and can easily be monitored in WinBUGS [69].

Similar to the Akaike Information Criterion, to which DIC reduces under a vague prior, DIC differences can be understood as a proxy of evidence for one model compared to the other, because they can be re-written as a quasi-evidential condition for the likelihood ratio: a model M1 is preferred by the data over M2 if and only if $\frac{P\left(y|\overline{\theta_1}\right)}{P\left(y|\overline{\theta_2}\right)}>\exp \left({p}_{D_1-}{p}_{D_2}\right)$, whereby $\overline{\theta_1}$ and $\overline{\theta_2}$ denote the posterior mean parameter estimates, and ${p}_{D_1}$ and ${p}_{D_2}$ the effective number of parameters of model 1 and 2, respectively.^5^ We note, however, that the likelihood $\frac{P\left(y|\overline{\theta_1}\right)}{P\left(y|\overline{\theta_2}\right)}$ is not invariant against reparametrization [67] and therefore disqualifies as a genuine evidence function [70]; that is why we call DIC differences only a proxy of evidence.

Another method of model comparison could be based on choosing the model with minimum entropy. By using the analytical expression for the entropy of a normally distributed variable [71], in each model we plugged in the posterior point estimate of the variance, i.e., its median ${\overline{\sigma}}^2$, to derive a representative estimate of the differential entropy of *θ*_*i*,*j*_ as $\overline{H}\left({\theta}_{i,j}|{\overline{\sigma}}^2\right)=0.5\ \ln \left(2\pi e{\overline{\sigma}}^2\right)$.

## Results

### Main model outcomes

**Fig. 2:**
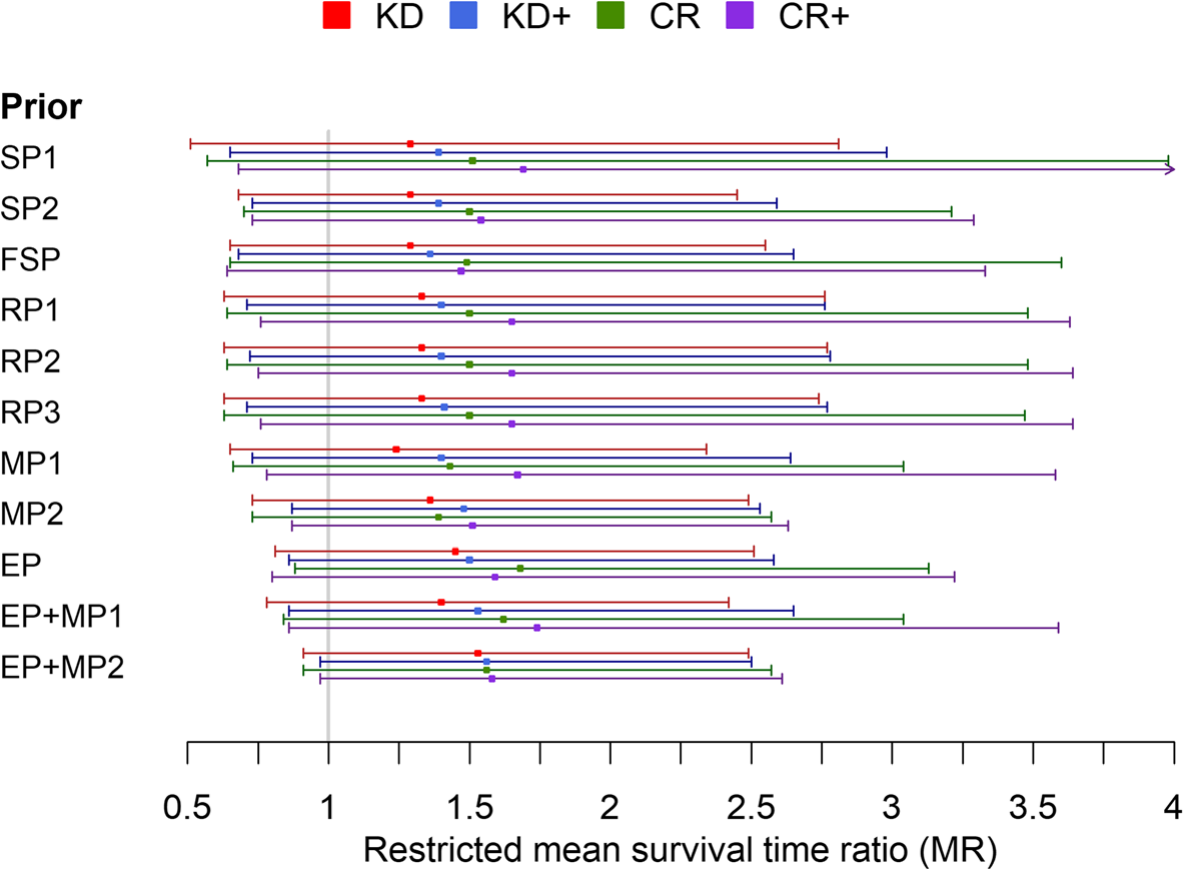
Posterior estimates of the restricted mean survival time ratio (MR) obtained with different prior specifications (see text for details). MR > 1 indicates longer survival with ketogenic therapy. CR/KD: Calorie restriction/ Ketogenic diet as monotherapy; CR+/KD+: Calorie restriction/ ketogenic diet combined with another therapy; EP: enthusiastic prior; FSP: Fundamentalist skeptical prior; MP: Mechanistic prior; RP: Relational prior; SP: Skeptical prior

The posterior MR estimates for humans obtained from fitting the model using different priors are given in Table 3 and displayed in Fig. 2. It can be seen that despite missing study data concerning the application of CR monotherapy to glioma patients, effect estimates have been obtained by utilizing information from other species and the various assumptions expressed in the priors. All posterior estimates indicated overall beneficial effects of ketogenic therapy, although the 95% CrIs display a relatively wide range of uncertainty which includes the range of non-beneficial effects. Noteworthy, this was also the case for the fundamentalist skeptical prior (FSP) which assumes tumor growth promoting effects of ketogenic therapy. In this case, the probability for a survival prolonging effect (MR > 1) was still > 75% for all interventions. The greatest uncertainties were obtained for the vague prior SP1 which has a large variance and ignores possible information that one species or intervention could contribute to others. On the other hand, modelling prior expectations based on mechanistic insights and published patient data reduced the uncertainties of posterior estimates. In general combinations of KDs or CR with other treatments resulted in greater effect estimates than each dietary intervention administered as monotherapy. The greatest probability of a benefit was obtained when adopting an enthusiastic prior based on previous case reports together with assuming correlations between KDs and CR and synergism between ketogenic therapy with other forms of treatment (prior EP + MP2); in this case, the probability for a survival prolongation was >90% for all four ketogenic interventions. However, compared to the prior expectation of MR = exp(0.3 + 0.336) = 1.89 for KD+/CR+ (because ${\mu}_{\alpha_1}=0.336$ in EP and ${\mu}_{\gamma_2}={\mu}_{\gamma_4}=0.3$ in MP2), the posterior estimates for combining KD or CR with other treatments indicated ≈30% less benefit, showing that these prior assumptions would have been too enthusiastic compared to the actual data. On the other hand, the data turned the prior belief in a detrimental effect of ketogenic therapy as modelled by an expected MR of 0.7 in the FSP into a ≳60% higher posterior estimate; thus, the fundamentalist sceptical prior assumption appeared even more unjustified than the enthusiastic one.

**Table 3:** Posterior estimates of the MR for humans

	KD	KD+	CR	CR+
Original study estimates	1.41 (0.61-2.21)	1.26 (0.77-1.75)	NA	1.75 (1.22-2.28)
Skeptical prior SP1	1.29 (0.58-2.81)	1.39 (0.65-2.98)	1.51 (0.57-3.98)	1.69 (0.68-4.22)
Skeptical prior SP2	1.29 (0.68-2.45)	1.39 (0.73-2.59)	1.50 (0.70-3.21)	1.54 (0.73-3.29)
Fundamentalist skeptical FSP	1.29 (0.65-2.55)	1.36 (0.68-2.65)	1.49 (0.65-3.6)	1.47 (0.64-3.33)
Relational priors RP1	1.33 (0.63-2.76)	1.40 (0.71-2.76)	1.50 (0.64-3.48)	1.65 (0.76-3.63)
Relational priors RP2	1.33 (0.63-2.77)	1.40 (0.72-2.78)	1.50 (0.64-3.48)	1.65 (0.75-3.64)
Relational priors RP3	1.33 (0.63-2.74)	1.41 (0.71-2.77)	1.50 (0.63-3.47)	1.65 (0.76-3.64)
Mechanistic prior MP1	1.24 (0.65-2.34)	1.40 (0.73-2.64)	1.43 (0.66-3.04)	1.67 (0.78-3.58)
Mechanistic prior MP2	1.36 (0.73-2.49)	1.48 (0.87-2.53)	1.39 (0.73-2.57)	1.51 (0.87-2.63)
Enthusiastic prior EP	1.45 (0.81-2.51)	1.50 (0.86-2.58)	1.68 (0.88-3.13)	1.59 (0.80-3.22)
Enthusiastic prior EP + MP1	1.40 (0.78-2.42)	1.53 (0.86-2.65)	1.62 (0.84-3.04)	1.74 (0.86-3.59)
Enthusiastic prior EP + MP2	1.53 (0.91-2.49)	1.56 (0.97-2.50)	1.56 (0.91-2.57)	1.58 (0.97-2.61)

The numbers give the median and 95% credible interval obtained with different prior distributions

The posterior estimates for *σ*, for which we had specified *σ*~*N*(0.5,0.01) a priori, were all within a range of [0.37,0.44] (Additional file 1: Table S1), indicating that the assumption of equal relative potency of the interventions was more acceptable in the model than suggested by its prior.

### Evidence for anti- versus pro-tumor effects

The DIC values (Additional file 1: Table S1) indicated that models assuming relational structures between species and interventions or incorporating mechanistic reasoning performed better than the skeptical models. The best performance was obtained for enthusiastic priors, in particular when expectations of anti-tumor effects where combined with mechanistic reasoning. However, there was no substantial difference between any two individual models, with the largest difference of ΔDIC = 1.858 observed between the enthusiastic EP + MP2 prior model and the skeptical SP1 prior model.

Comparing the models in terms of entropy also picked out the EP + MP2 prior model as the one to prefer, although entropy differences among the models where not strongly pronounced (Additional file 1: Table S1). This is expected since the entropy does not penalize sufficiently for model complexity, so that DIC is to be preferred [68].

### Sensitivity analysis

The sensitivity of the model to different prior assumptions was evaluated and results are given in the Additional file 1.

#### Sensitivity to the prior on *σ*

Posterior estimates of the MR for humans depending on the prior specification for *σ* are given in Additional file 1: Table S2, adopting the skeptical prior SP2 for *α* and *γ* in all cases. The posterior estimates for the MRs did not vary substantially with the choice of the prior for *σ*; however, the width of the posterior 95% CrIs and the point estimate for *σ* itself were quite sensitive to that choice. Consistent with the main analysis, the posterior estimates for *σ* were always closer to zero than their prior mean specifications, indicating better acceptability for the assumption of equal relative potency than specified a priori.

#### Removal of individual studies

We assessed the impact of removing experiments that basically replicated other experiments: We removed the studies of Stafford et al. [47] and Lussier et al. [53], because they used the same tumor model as Abdelwahab et al. [48] (the latter was kept because it tested both the KD as monotherapy and in combination with radiotherapy). We also removed the study by Marsh et al. [46] which used the same tumor model that had already been used by Zhou et al. [45]. Except for slightly higher effect estimates for KD and CR monotherapy, this had a negligible impact on the results (Additional file 1: Table S3).

#### Removal of study by Rieger et al.

**Fig. 3:**
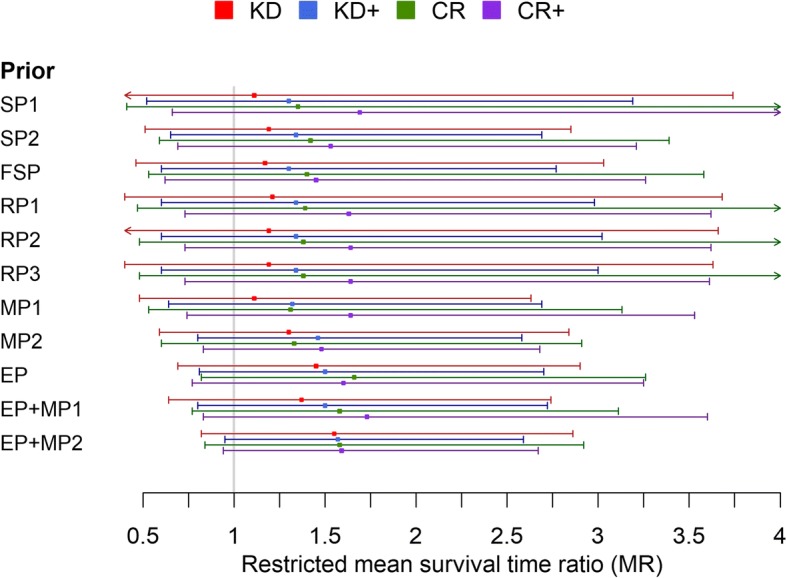
Posterior estimates of the restricted mean survival time ratio (MR) obtained with different prior specifications, but without using the datum from Rieger et al. This figure should be compared to Fig. 2

Rieger et al. [50] compared progression free survival between patients in stable ketosis and those without stable ketosis despite prescription of a KD. Since this control group differs from the usual standard diet groups, we tested the effect of removing this study’s MR estimate from the analysis. The result is shown in Fig. 3 and tabulated in Additional file 1: Table S4. As expected, removal of the Rieger et al. data led to increased uncertainty in the posterior estimate of the MR for KD and – to a lesser extent – KD+ interventions. In addition, a visibly lower KD effect estimate was obtained for most of the priors except the enthusiastic ones. Nevertheless, even with the sceptical priors the bulk of the MR posterior density indicated a beneficial effect of ketogenic therapy.

## Discussion

Clinical and biomedical inference is inductive in nature [24]. In his “principle of total evidence”, the philosopher Rudolf Carnap pointed out the importance of considering the totality of evidence for testing (confirming) hypotheses through inductive reasoning [72]. By constituting a rigid evidence hierarchy, EBM seems to violate this principle, because evidence of “lesser dignity”, such as mechanistic studies, case reports or uncontrolled cohort studies are discarded from the evidence synthesis model as soon as “better” evidence from RCTs becomes available [19]. The eliminatory process of reducing information down to only a few RCTs tends to create comparatively small sets of homogenous studies. Depending on the number of studies, their quality and the number of participants involved, the pooled confidence intervals can become comparatively small, but at the expense of generalizing to a broader patient population (external validity). Some proponents of RCTS have even claimed that only “*the process of randomization makes it possible to ascribe a probability distribution to the difference in outcome between treatment groups receiving equally effective treatments and thus to assign ‘significance levels’ to observed differences*” [73], implying that randomization is essential to perform significance tests and estimate confidence intervals in the first place. This argument suffers from the serious problem that the target population from which the study groups are supposed to be random samples cannot be clearly defined [24, 74]. Furthermore, confidence intervals can in practise also be derived for non-randomized studies while accounting for confounders. Yet, the fundamental importance of randomization has remained in the frequentist paradigm, so that non-randomized studies are usually excluded from standard meta-analyses. Several statisticians and philosophers have cautioned against this approach, such as Steven Goodman who argued that “*the methods of statistical inference in current use are not ‘evidence-based’ and thus have contributed to a widespread misperception … [that] has damaged the quality of scientific reasoning and discourse, primarily by making it difficult to understand how the strength of evidence in a particular study can be related to and combined with the strength of other evidence (from other laboratory or clinical studies, scientific reasoning, or clinical experience)*” [25]. As Goodman and others point out, Bayesian methods do not have these problems as they naturally provide a measure of evidence in form of the Bayes factor or likelihood ratio [32, 75]. From a Bayesian perspective, there is also a less important role for randomization [74], and methods to synthesize different study types while accounting for their variability with respect to effect estimates exist [37]. Hence, the Bayesian approach is the only one allowing the integration of a more varied dataset from different study designs, appearing more able to respect Carnap’s total evidence requirement as well as common sense. This however not only introduces more studies, but also more sources of bias and therefore more heterogeneity which will likely lead to larger credibility intervals around the overall effect estimate, if those different datasets will not converge. But if they converge we can expect a stronger effect with higher credibility than an eliminatory process can provide.

Thus the Bayesian approach appears ideally suited for a circular view of evidence whose methodological principles have been described formerly [31]. Our goal in this paper was to carry out a Bayesian evidence synthesis, taking ketogenic therapy for GBM as an example for which according to EBM no “real evidence” would yet exist. By accounting for mechanisms, mainly in form of the biological hypothesis that the effect ratio between two different ketogenic interventions would be preserved across species (equal relative potency of interventions), we constructed a model which allowed us to combine evidence from human and animal studies and account for different further assumptions relating to external knowledge. While the model builds on previous work and is similar in structure to the models by DuMouchel and Harris [34] and Jones et al. [39], it extends these models by exemplarily showing how a variety of prior beliefs or information could be accounted for.

As a main result, our analysis yields an overall beneficial effect of ketogenic therapy in HGG patients, although the uncertainties were too large to render these effects “significant” in a classical frequentist sense. An overall beneficial effect was also obtained even when a fundamentalist skeptical prior was used that assumes that ketogenic therapy would have the effect of promoting tumor growth and shortening survival. Even in this case, the posterior MR distribution supported beneficial effects of KDs and CR with more than 75 and 80% probability, respectively. Therefore the data provide some justification for the hypothesis of an overall life-prolonging and anti-tumor effect of ketogenic therapy in HGG. However, if the DIC differences are taken as proxies for an evidence measure via the likelihood ratio, we must conclude that more positive outcome data would be needed to also provide strong evidence for this hypothesis (compared e.g. to the hypothesis of pro-tumor effects).

The results of our analysis are in line with the qualitative systematic review by Winter et al. [7] which concludes that a “*substantial amount of preclinical literature demonstrates [ketogenic therapy] efficacy and safety in model systems of malignant glioma*” and that the current clinical studies “*suggest [ketogenic therapy] is safe and feasible in brain cancer patients, with some encouraging indications of potential anti-neoplastic effects and clinical utility in malignant glioma treatment*”. Our study indicates a greater benefit when ketogenic therapy is combined with other treatment modalities, but at the same time our analysis is limited by only a small percentage of studies investigating such combinations. Furthermore, this fact prohibited a more detailed analysis differentiating between different types of concomitant treatment. Nevertheless, synergistic effects of ketogenic therapy with other treatment modalities have also been found in non-HGG tumor models. Examples include combinations of KD and hyperbaric oxygen [76], KD and radio-chemotherapy [77], CR and radiotherapy [78], or KD/CR and chemotherapy [79]; the underlying mechanisms have been reviewed in [12–14, 59].

Methodologically, the strength of our Bayesian evidence synthesis is that the individual survival data it is based on are almost exactly known: some have been retrieved from the study authors, while most come from direct read offs of the Kaplan-Meier plots (always double-checking by computing survival estimates and comparing them to the values read off the graphs). Epistemologically, the strength of our model is that it is based on a biological hypothesis (of equal relative potencies of interventions across species) and that it incorporates animal studies that not only provide statistical data (which by their nature are non-explanatory) but also a connection between these statistical data and mechanistic explanation of how ketogenic therapy influences tumor biology. Furthermore, we have incorporated mechanistic reasoning and case reports through priors and shown that this improves the model fit, providing a link between explanatory qualitative research and statistical associations, both of which are required to establish causation [80]. In this way our evidence synthesis overcomes some of the limitations of conventional meta-analysis which typically excludes qualitative and experimental empirical research (such as preclinical studies and case reports) and therefore lacks explanatory power [81].

Nevertheless, while our statistical model is mainly concerned with a quantitative prediction of the survival benefit of HGG patients in response to ketogenic therapy, it falls short of providing context-dependent explanations such as why and under which conditions we would observe a survival prolongation in a given patient receiving a certain ketogenic therapy. Such inquiries are typical for realist reviews, which are rooted in the social sciences [82] but increasingly used for evaluating complex heath care interventions [83]. By connecting mechanisms with statistical quantities as described above, we consider our analysis complementary to such qualitative realist syntheses, which could help clinicians with mechanistic reasoning and guide discussions with patients in regards to the risk and rationale of implementing ketogenic therapy in light of the totality of current evidence. Other approaches in areas in which according to EBM only very low level evidence exists try to use expert opinion and consensus together with mechanistic reasoning and the available clinical data; there are even some cases where evidence of mechanisms becomes more important than statistical evidence for establishing a causal claim of treatment efficacy. Such an example related to our topic here is the treatment of the rare disease glucose transporter 1 (GLUT1) deficiency syndrome using KDs: despite only “low level” evidence available, a recent consensus guideline recommends KDs as the treatment of choice for GLUT1 deficiency syndrome mainly based on the physiological mechanisms that ketone bodies are able to cross the blood-brain barrier independent from GLUT1, providing an alternative fuel for the brain instead of glucose [84].

Given the results of our analysis, we try to briefly answer the “what should we do?” question which is one of four fundamental epistemological questions and relates to the acceptance of a hypothesis (Additional file 1: Appendix A) − in our case that ketogenic therapy prolongs survival in HGG patients. As Bayesians, we contend that in a decision situation one ought to accept the hypothesis which has a higher expected utility than any other. Given the low extra costs of ketogenic therapy, its low probability of severe side effects [85] and great potential to reduce epileptic seizures [84, 86], which are a common side effect of HGG, we would expect some utility for patients who wish to utilize ketogenic therapy, so that their physicians should not be discouraged from supporting them in this attempt. Physicians, however, should keep the limitations of our analysis and the studies it is based on in mind. First, as already noted, the number of studies as well as the number of human and animal subjects treated therein is very limited, as is the number of (animal and human) studies investigating different combinations of ketogenic and other therapies. Thus, there are many possible contexts in which the approach has never been evaluated. Second, all of the included studies suffer from various forms of bias such as reporting, performance and selection bias in the animal studies [43] and selection and performance bias in the human studies due to missing randomization [19]. The risk of bias in all studies was judged as high, but similar enough to not account for it in the analysis.^6^ Acknowledging the various forms of bias in the studies utilized in our model, we judge the real uncertainties of our overall results as higher than estimated. Third, our model depends on the plausibility of the equal relative potency assumption. The fact that the posterior estimate of *σ*, which measures the accuracy of this assumption, was always closer to zero than its prior at least indicates some plausibility of equal relative potency of the intervention across species. Fourth, the different priors we have tested are all more or less subjective and in no way exhaustive as a representation of what different agents or experts could belief about the expected effects of ketogenic therapy in HGG patients. Nevertheless, by testing a total of eleven different prior assumptions, we have covered a range of varying beliefs regarding beneficial effects of ketogenic therapy including very skeptical as well as enthusiastic expectations. The fact that no substantial DIC differences existed for all these priors indicates that the posterior estimates were much more sensitive to the data than the priors.

Finally, we have shown that this approach, integrating a Bayesian analysis across different types of studies and designs is not only feasible, but yields useful clinical insights that would be difficult to glean from standard modes of analysis and reviews. Thus, we have provided a proof of concept analysis that makes the circular view of evidence not only more realistic than the hierarchical one, but also provides EBM with an important new tool to improve the methodology of synthesizing evidence in such a modality.

## Conclusions

In conclusion, we have found an overall survival-prolonging effect of ketogenic therapy in HGG patients by combining evidence from both human and animal studies. These findings are in line with the general notion of anti-tumor effects of ketogenic therapy as summarized in several expert revqiews [5, 16, 87–89]. However, our analysis is the first to provide quantitative estimates for the expected survival prolongation in the form of MRs, and one of only a few that attempts to extrapolate quantitative results from preclinical studies to humans. Our approach is best compatible with a circular instead of hierarchical view of evidence and complementary to more qualitative research-oriented reviews mainly concerned with the mechanisms and context-dependency of intervention effects. A limitation of our model consists in the low number of and high risk of bias in the human and animal data it incorporates, which would make the uncertainties of our overall results larger than estimated. However, it will be straightforward to update our model once new and more precise data from planned or currently running clinical trials (e.g. [90]) become available.

## Additional file

Additional file 1:  Philosophical concepts (Appendix A), sample BUGS code (Appendix B) and supplementary tables (Appendix C). (PDF 368 kb)

## Abbreviations

CR: Calorie restriction
EBM: Evidence Based Medicine
EP: enthusiastic prior
FSP: Fundamentalist skeptical prior
GBM: glioblastoma multiforme
HGG: High grade glioma
KD: Ketogenic diet
MP: Mechanistic prior
RP: Relational prior
SP: Skeptical prior

## Footnotes

1 Mechanistic reasoning can be defined according to Howick as involving “an inference from mechanisms to claims that an intervention produces a patient-relevant outcome. Such reasoning will involve an inferential chain linking the intervention … with a clinical outcome (such as mortality)” [19].

2 Concerning the general inflexibility of frequentist methods, Jordi Vallverdú writes: “Perhaps one of the most unnoticed but deep problems of frequentist techniques has been the strict perspective about the range of available statistic tools as well as sometimes naïve perspective about how real science is performed … Then, when Bayesian methods were reintroduced, they spread fast among scientists, not for philosophical reasons but for practical ones” ([91], page 73).

3 During data extraction, we noticed an inconsistency between the number of mice needed to make sense of the Kaplan-Meier survival percentages and those given in the figure caption of Figure 6 in De Feyter et al. [52]. The issue has been resolved with the authors and the correct numbers are used in our analysis.

4 The term “fundamentalist skeptic” was used by Klement [44] to describe skeptics of ketogenic therapy who believe that this therapy is dangerous and able to promote tumor growth despite a lack of evidential support indicating otherwise. The term was borrowed from the astronomer-philosopher Milan M. Ćirković [92].

5 The derivation is analogous to that for AIC (see Forster & Sober [93]): Model M1 is favored over M2 if and only if DIC1 − DIC2 < 0, so that
$$ {\displaystyle \begin{array}{l}-2\left[\log \mathrm{p}\left(y|\overline{\theta_1}\right)-\log \mathrm{p}\left(y|\overline{\theta_2}\right)-\left({p}_{D_1-}{p}_{D_2}\right)\right]<0\\ {}\Rightarrow \log \left(\frac{\mathrm{p}\left(y|\overline{\theta_1}\right)}{\mathrm{p}\left(y|\overline{\theta_2}\right)}\right)>{p}_{D_1-}{p}_{D_2}\\ {}\Rightarrow \frac{\mathrm{p}\left(y|\overline{\theta_1}\right)}{\mathrm{p}\left(y|\overline{\theta_2}\right)}>\exp \left({p}_{D_1-}{p}_{D_2}\right).\end{array}} $$

6 For methods to incorporate risk of bias of certain study types into a Bayesian evidence synthesis see e.g. Welton et al. [37].

## References

[1] Stupp R, Masen WP, van den Bent MJ, Weller M, Fisher B, Taphoorn MJB (2005). Radiotherapy plus concomitant and adjuvant Temozolomide for glioblastoma. N Engl J Med.

[2] Adeberg S, Bernhardt D, Ben HS, Uhl M, Paul A, Bougatf N, et al. Sequential proton boost after standard chemoradiation for high-grade glioma. Radiother Oncol. 2017;125:266–72. 10.1016/j.radonc.2017.09.040 29050959

[3] Seyfried TN, Kiebish M, Mukherjee P, Marsh J. Targeting energy metabolism in brain cancer with calorically restricted ketogenic diets. Epilepsia. 2008;49(Suppl 8):114–6. 10.1111/j.1528-1167.2008.01853.x 19049606

[4] Maroon JC, Seyfried TN, Donohue JP, Bost J (2015). The role of metabolic therapy in treating glioblastoma multiforme. Surg Neurol Int.

[5] Woolf EC, Scheck AC (2015). The ketogenic diet for the treatment of malignant glioma. J Lipid Res.

[6] Woolf EC, Syed N, Scheck AC (2016). Tumor metabolism, the ketogenic diet and β-Hydroxybutyrate: novel approaches to adjuvant brain tumor therapy. Front Mol Neurosci.

[7] Winter SF, Loebel F, Dietrich J (2017). Role of ketogenic metabolic therapy in malignant glioma: a systematic review. Crit Rev Oncol Hematol.

[8] Tisdale MJ, Brennan RA (1983). Loss of acetoacetate coenzyme a transferase activity in tumours of peripheral tissues. Br J Cancer.

[9] Maurer GD, Brucker DP, Bähr O, Harter PN, Hattingen E, Walenta S (2011). Differential utilization of ketone bodies by neurons and glioma cell lines: a rationale for ketogenic diet as experimental glioma therapy. BMC Cancer.

[10] Chang HT, Olson LK, Schwartz KA (2013). Ketolytic and glycolytic enzymatic expression profiles in malignant gliomas: implication for ketogenic diet therapy. Nutr Metab (Lond).

[11] Schwartz K, Chang HT, Nikolai M, Pernicone J, Rhee S, Olson K (2015). Treatment of glioma patients with ketogenic diets: report of two cases treated with an IRB-approved energy-restricted ketogenic diet protocol and review of the literature. Cancer Metab.

[12] Seyfried TN, Yu G, Maroon JC, D’Agostino DP (2017). Press-pulse: a novel therapeutic strategy for the metabolic management of cancer. Nutr Metab (Lond).

[13] Klement RJ. The influence of ketogenic therapy on the 5 R’s of radiobiology. Int J Radiat Biol. 2017; 10.1080/09553002.2017.1380330. 10.1080/09553002.2017.1380330 28905672

[14] Klement RJ. Fasting, fats, and physics: combining ketogenic and radiation therapy against Cancer. Complement Med Res. 2017. 10.1159/000484045 29130953

[15] Klement RJ (2014). Restricting carbohydrates to fight head and neck cancer—is this realistic?. Cancer Biol Med.

[16] Hyde PN, Lustberg MB, Miller VJ, LaFountain RA, Volek JS (2017). Pleiotropic effects of nutritional ketosis: conceptual framework for keto-adaptation as a breast cancer therapy. Cancer Treat Res Commun.

[17] Tella SH, Kommalapati A, Esquivel MA, Correa R (2017). Potential role of metabolic intervention in the Management of Advanced Differentiated Thyroid Cancer. Front Oncol.

[18] Worrall J (2007). Evidence in medicine and evidence-based medicine. Philos Compass.

[19] Howick J (2011). The philosophy of evidence-based medicine. 1st edition.

[20] Andersen H (2012). Mechanisms: what are they evidence for in evidence-based medicine?. J Eval Clin Pract.

[21] Greenhalgh T, Howick J, Maskrey N, Evidence Based Medicine Renaissance Group (2014). Evidence based medicine: a movement in crisis?. BMJ.

[22] Martin-McGill KJ, Marson AG, Smith CT, Jenkinson MD. The Modified Ketogenic Diet in Adults with Glioblastoma: An Evaluation of Feasibility and Deliverability within the National Health Service. Nutr Cancer. 2018;70:643–49. 10.1080/01635581.2018.1460677 29668317

[23] Gonder U (2017). Article on ketogenic dietary regimes for cancer highly misleading. Med Oncol.

[24] Thompson RP, Upshur REG (2017). Philosophy of medicine.

[25] Goodman SN (1999). Toward evidence-based medical statistics. 1: the P value fallacy. Ann Intern Med.

[26] Berry DA. A case for Bayesianism in clinical trials. Stat Med. 1993;12:1377–93. 10.1002/sim.4780121504 8248653

[27] Sutton AJ, Abrams KR (2001). Bayesian methods in meta-analysis and evidence synthesis. Stat Methods Med Res.

[28] Cartwright N, Howick J (2009). Evidence-based policy: where is our theory of evidence?. J Child Serv.

[29] Horton R (2015). Offline: what is medicine’s 5 sigma?. Lancet.

[30] Bailer-Jones CAL (2017). Frequentist hypothesis testing. Practical Bayesian inference. A primer for physical scientists.

[31] Walach H, Falkenberg T, Fønnebø V, Lewith G, Jonas WB (2006). Circular instead of hierarchical: methodological principles for the evaluation of complex interventions. BMC Med Res Methodol.

[32] Bandyopadhyay PS, Brittan G Jr, Taper ML. Belief, Evidence, and Uncertainty: problems of epistemic inference. Basel: Springer International Publishing; 2016.

[33] Walach H, Loef M (2015). Using a matrix-analytical approach to synthesizing evidence solved incompatibility problem in the hierarchy of evidence. J Clin Epidemiol.

[34] DuMouchel WH, Harris JE (1981). Bayes methods for combining the results of Cancer studies in humans and other species. J Am Stat Assoc.

[35] Prevost TC, Abrams KR, Jones DR (2000). Hierarchical models in generalized synthesis of evidence: an example based on studies of breast cancer screening. Stat Med.

[36] Woods BS, Hawkins N, Scott DA (2010). Network meta-analysis on the log-hazard scale, combining count and hazard ratio statistics accounting for multi-arm trials: a tutorial. BMC Med Res Methodol.

[37] Welton NJ, Sutton AJ, Cooper NJ, Abrams KR, Ades AE (2012). Evidence synthesis for decision making in healthcare.

[38] DuMouchel W, Groër PG (1989). A Bayesian methodology for scaling radiation studies from animals to man. Health Phys.

[39] Jones DR, Peters JL, Rushton L, Sutton AJ, Abrams KR (2009). Interspecies extrapolation in environmental exposure standard setting: a Bayesian synthesis approach. Regul Toxicol Pharmacol.

[40] Spruance SL, Reid JE, Grace M, Samore M (2004). Hazard ratio in clinical trials. Antimicrob Agents Chemother.

[41] Royston P, Parmar MK (2013). Restricted mean survival time: an alternative to the hazard ratio for the design and analysis of randomized trials with a time-to-event outcome. BMC Med Res Methodol.

[42] Uno H, Claggett B, Tian L, Inoue E, Gallo P, Miyata T (2014). Moving beyond the hazard ratio in quantifying the between-group difference in survival analysis. J Clin Oncol.

[43] Klement RJ, Champ CE, Otto C, Kämmerer U (2016). Anti-tumor effects of ketogenic diets in mice: a meta-analysis. PLoS One.

[44] Klement RJ (2017). Beneficial effects of ketogenic diets for cancer patients: a realist review with focus on evidence and confirmation. Med Oncol.

[45] Zhou W, Mukherjee P, Kiebish MA, Markis WT, Mantis JG, Seyfried TN. The calorically restricted ketogenic diet, an effective alternative therapy for malignant brain cancer. Nutr Metab (Lond). 2007;4:5. 10.1186/1743-7075-4-5 PMC1819381 17313687

[46] Marsh J, Mukherjee P, Seyfried TN (2008). Akt-dependent proapoptotic effects of dietary restriction on late-stage management of a phosphatase and Tensin homologue/ tuberous sclerosis complex 2-deficient mouse astrocytoma. Clin Cancer Res.

[47] Stafford P, Abdelwahab MG, Kim DY, Preul MC, Rho JM, Scheck AC (2010). The ketogenic diet reverses gene expression patterns and reduces reactive oxygen species levels when used as an adjuvant therapy for glioma. Nutr Metab (Lond).

[48] Abdelwahab MG, Fenton KE, Preul MC, Rho JM, Lynch A, Stafford P (2012). The ketogenic diet is an effective adjuvant to radiation therapy for the treatment of malignant glioma. PLoS One.

[49] Jiang Y-S, Wang F-R (2013). Caloric restriction reduces edema and prolongs survival in a mouse glioma model. J Neuro-Oncol.

[50] Rieger J, Bähr O, Maurer GD, Hattingen E, Franz K, Brucker D (2014). ERGO: a pilot study of ketogenic diet in recurrent glioblastoma. Int J Oncol.

[51] Martuscello RT, Vedam-Mai V, McCarthy DJ, Schmoll ME, Jundi MA, Louviere CD (2016). A supplemented high-fat low-carbohydrate diet for the treatment of glioblastoma. Clin Cancer Res.

[52] De Feyter HM, Behar KL, Rao JU, Madden-Hennessey K, Ip KL, Hyder F (2016). A ketogenic diet increases transport and oxidation of ketone bodies in RG2 and 9L gliomas without affecting tumor growth. Neuro-Oncology.

[53] Lussier DM, Woolf EC, Johnson JL, Brooks KS, Blattman JN, Scheck AC (2016). Enhanced immunity in a mouse model of malignant glioma is mediated by a therapeutic ketogenic diet. BMC Cancer.

[54] Han L, Zhang J, Zhang P, Han X, Bi Z, Li J (2014). Perspective research of the influence of caloric restriction combined with psychotherapy and chemotherapy. Zhonghua Yi Xue Za Zhi.

[55] Bandyopadhyay PS, Dey D, Upadhyay SK (2007). Why Bayesianism? A primer on a probabilistic philosophy of science. Bayesian statistics and its applications.

[56] Klement RJ (2013). Calorie or carbohydrate restriction? The ketogenic diet as another option for supportive Cancer treatment. Oncologist.

[57] Mahoney LB, Denny CA, Seyfried TN (2006). Calorie restriction in C57BL/6J mice mimics therapeutic fasting in humans. Lipids Health Dis.

[58] Chandrasekera PC, Pippin JJ (2013). Of rodents and men: species-specific glucose regulation and type 2 diabetes research. ALTEX.

[59] Klement RJ, Champ CE (2014). Calories, carbohydrates, and cancer therapy with radiation: exploiting the five R’s through dietary manipulation. Cancer Metastasis Rev.

[60] Nebeling L, Miraldi F, Shurin S, Lerner E (1995). Effects of a ketogenic diet on tumor metabolism and nutritional status in pediatric oncology patients: two case reports. J Am Coll Nutr.

[61] Moore K. Using the restricted ketogenic diet for brain cancer management: comments from neuro-oncologist. In: Seyfried TN, editor. Cancer as a metabolic disease: on the origin, management, and prevention of Cancer. 1st ed. Hoboken: Wiley; 2012. p. 397–400.

[62] Abdelbary M, Elsakka A, Salah H, Mokhtar W, Abdelzaher E. Does metabolic Management of Gliomas Using Restricted Ketogenic Diet Combined with hyperbaric oxygen therapy (HBOT) improve clinical outcome and reduce epileptic risk? Poster presented at the 2nd Conference on Nutritional Ketosis and Metabolic Therapeutics. Tampa, FL; 2017.

[63] Zuccoli G, Marcello N, Pisanello A, Servadei F, Vaccaro S, Mukherjee P (2010). Metabolic management of glioblastoma multiforme using standard therapy together with a restricted ketogenic diet: case report. Nutr Metab.

[64] Champ CE, Palmer JD, Volek JS, Werner-Wasik M, Andrews DW, Evans JJ (2014). Targeting metabolism with a ketogenic diet during the treatment of glioblastoma multiforme. J Neuro-Oncol.

[65] Artzi M, Liberman G, Vaisman N, Bokstein F, Vitinshtein F, Aizenstein O (2017). Changes in cerebral metabolism during ketogenic diet in patients with primary brain tumors: 1H-MRS study. J Neuro-Oncol.

[66] Spiegelhalter DJ, Best NG, Carlin BP, van der Linde A (2002). Bayesian measures of model complexity and fit. J R Stat Soc Ser B Stat Methodol.

[67] Spiegelhalter DJ, Best NG, Carlin BP, van der Linde A (2014). The deviance information criterion: 12 years on. J R Stat Soc Ser B Stat Methodol.

[68] van der Linde A (2005). DIC in variable selection. Stat Neerl.

[69] Lunn D, Jackson C, Best N, Thomas A, Spiegelhalter D. The BUGS book: a practical introduction to Bayesian analysis. 1st ed. Boca Raton, FL: Chapman and Hall/CRC; 2012.

[70] Lele SR, Taper ML, Lele SR (2004). Evidence functions and the optimality of the law of likelihood. The nature of scientific evidence.

[71] Verdugo Lazo ACG, Rathie PN (1978). On the entropy of continuous probability distributions. IEEE Trans Inf Theory.

[72] Carnap R (1947). On the application of inductive logic. Philos Phenomenol Res.

[73] Byar DP, Simon RM, Friedewald WT, Schlesselman JJ, DeMets DL, Ellenberg JH (1976). Randomized clinical trials. Perspectives on some recent ideas. N Engl J Med.

[74] Urbach P (1993). The value of randomization and control in clinical trials. Stat Med.

[75] Goodman SN. Toward evidence-based medical statistics. 2: the Bayes factor. Ann Intern Med. 1999;130:1005–13. 10.7326/0003-4819-130-12-199906150-00019 10383350

[76] Poff AM, Ari C, Seyfried TN, D’Agostino DP (2013). The ketogenic diet and hyperbaric oxygen therapy prolong survival in mice with systemic metastatic Cancer. PLoS One.

[77] Allen BG, Bhatia SK, Buatti JM, Brandt KE, Lindholm KE, Button AM (2013). Ketogenic diets enhance oxidative stress and radio-chemo-therapy responses in lung Cancer xenografts. Clin Cancer Res.

[78] Saleh AD, Simone BA, Savage J, Sano Y, Jin L, Champ C (2013). Caloric restriction augments radiation efficacy in breast cancer. Cell Cycle.

[79] Morscher RJ, Aminzadeh-Gohari S, Hauser-Kronberger C, Feichtinger RG, Sperl W, Kofler B (2016). Combination of metronomic cyclophosphamide and dietary intervention inhibits neuroblastoma growth in a CD1-nu mouse model. Oncotarget.

[80] Russo F, Williamson J (2007). Interpreting causality in the health sciences. Int Stud Philos Sci.

[81] Brannan MJ, Fleetwood S, O’Mahoney J, Vincent S (2017). Critical essay: meta-analysis: a critical realist critique and alternative. Hum Relations.

[82] Pawson R, Greenhalgh T, Harvey G, Walshe K (2005). Realist review – a new method of systematic review designed for complex policy interventions. J Health Serv Res Policy.

[83] Wong G, Greenhalgh T, Westhorp G, Buckingham J, Pawson R, Pawson R (2013). RAMESES publication standards: realist syntheses. BMC Med.

[84] Kossoff EH, Zupec-Kania BA (2018). Optimal clinical management of children receiving the ketogenic diet: recommendations of the international ketogenic diet study group. Epilepsia Open.

[85] Klement RJ, Feinman RD, Gross EC, Champ CE, D’Agostino DP, Fine EJ (2017). Need for new review of article on ketogenic dietary regimes for cancer patients. Med Oncol.

[86] McDonald TJW, Cervenka MC (2017). Ketogenic diets for adults with highly refractory epilepsy. Epilepsy Curr.

[87] Paoli A, Rubini A, Volek JS, Grimaldi KA (2013). Beyond weight loss: a review of the therapeutic uses of very-low-carbohydrate (ketogenic) diets. Eur J Clin Nutr.

[88] Allen BG, Bhatia SK, Anderson CM, Eichenberger-Gilmore JM, Sibenaller ZA, Mapuskar KA (2014). Ketogenic diets as an adjuvant cancer therapy: history and potential mechanism. Redox Biol.

[89] Seyfried TN, Flores RE, Poff AM, D’Agostino DP (2014). Cancer as a metabolic disease: implications for novel therapeutics. Carcinogenesis.

[90] Martin-McGill KJ, Marson AG, Tudur Smith C, Jenkinson MD (2017). Ketogenic diets as an adjuvant therapy in glioblastoma (the KEATING trial): study protocol for a randomised pilot study. Pilot Feasibility Stud.

[91] Vallverdú J. Bayesians versus frequentists. 1st ed. Berlin: Springer Berlin Heidelberg; 2016.

[92] Ćirković MM (2013). Who are the SETI sceptics?. Acta Astronaut.

[93] Forster M, Sober E, Taper ML, Lele SR (2004). Why likelihood?. The nature of scientific evidence.

