# Supplementary material for: Application of Bayesian evidence synthesis to modelling the effect of ketogenic therapy on survival of high grade glioma patients
Source: Theor Biol Med Model. 2018 Aug 20;15:12. doi: 10.1186/s12976-018-0084-y (PMC6100754; doi:10.1186/s12976-018-0084-y)
Supplement: Supplementary file 1 — Philosophical concepts (Appendix A), sample BUGS code (Appendix B) and supplementary tables (Appendix C). (PDF 368 kb) [file 12976_2018_84_MOESM1_ESM.pdf]

Appendix A. Philosophical Foundation for a Bayesian Synthesis

The purpose of this appendix is to provide a foundation for our version of Bayesianism, which, in turn, both depends on distinguishing between four questions and subsequently providing accounts for each of them. We will begin with these four questions as they provide a niche for understanding the current debate among paradigms in the foundations of scientific inference.

Scientific Inference and Four Questions

Philosophy of science is, among other things, a careful reflection on the scientific methodology that informs both discovering and justifying theories. The tenability of scientific theories turns crucially on making inferences based on data, well-supported theories, and auxiliaries. A comprehensive understanding of scientific theories thus requires an understanding of scientific inference, broadly construed. However, several epistemological issues need to be distinguished in order to appreciate the proper relationship between the tenability of scientific theories and inference. We will discuss the significance of these issues/questions by borrowing an insight from Richard Royall (Royall 1997; Royall 2004). However, as it will become clearer, our work, in turn, provides a unified Bayesian response to four question including Royall.

Consider two hypotheses: H, representing that a patient with partially resected glioblastoma multiforme experiences tumor progression after radio-chemotherapy, and ~H, its denial. Assume that an MRI scan, which is administered as a follow-up test 3 months after therapy, comes out positive for tumor progression. Based on this simple scenario, one could pose four types of question that underline the epistemological issues at stake:

(i) Given the datum, what should we *believe* about H and to what degree?

(ii) Does the datum provide strong *evidence* for H against its alternative ~H?

(iii) What does the datum tell us about the *predictive accuracy* of the hypothesis?

(iv) Given the datum, what should we *do*?

We call the first question the belief or confirmation question, the second the evidence question, the third the prediction question, the fourth one the decision question. These four questions are pre-theoretical and statistical paradigm-neutral; yet they require some statistical/probabilistic tools for their articulation.

Confirmation and Evidence

We have developed two distinct accounts to answer the first two types of questions. The first is an account of belief/confirmation, the second of evidence. Many Bayesians interpret confirmation relations in various ways. For us, an account of confirmation explicates a relation, C(D,H,B) among data D, hypothesis H, and the agent's background knowledge B. For Bayesians, degrees of belief need to be fine-grained. A satisfactory Bayesian account of confirmation, according to us, should be able to capture this notion of degree of belief. In formal terms: D confirms H if and only if $Pr(H|D) > Pr(H)$.

The posterior/prior probability of H could vary between 0 and 1. Confirmation becomes strong or weak depending on how great the difference is between the posterior probability, Pr(H|D), and the prior probability of the hypothesis, Pr(H). Pr(H|D) represents an agent's degree of belief in the hypothesis after the data are accumulated. Pr(H) stands for an agent's degree of belief in the hypothesis before the data for the hypothesis have been acquired. The likelihood function, Pr(D|H), provides an answer to the question "how likely are the data given the hypothesis"? Pr(D) is the marginal probability of the data averaged over the hypothesis being true or false. The relationships between these terms, Pr(H|D), Pr(H), and Pr(D|H), and Pr(D) are succinctly captured in Bayes' theorem: $Pr(H|D) = [Pr(H) \times Pr(D|H)]/Pr(D) > 0$.

While this account of confirmation is concerned with belief in a single hypothesis, our account of evidence compares the merits of two simple statistical hypotheses, H1 and H2 (or ~H1) relative to the data D, auxiliaries A, and background information B. However, because evidence is not a belief relation, but a likelihood ratio, it need not satisfy the probability calculus. Bayesians use the Bayes factor (BF) to make this comparison, while others use the likelihood ratio (LR) or other functions designed to measure evidence. $BF = [Pr(D|H1,A1\&B)]/ [Pr(D|H2,A2\&B)]$ is called the Bayes factor in favor of H1, given A1 and B. For hypothesis under which there are unknown parameters θ, the densities[1] Pr(D|H,A&B) are obtained by integrating over the parameter space, so that $Pr(D|H,A\&B) = \int Pr(D|\theta,H,A\&B)\pi(\theta|H,A\&B)d\theta$ (Kass & Raftery 1995). For simple statistical hypotheses with no free parameters, the Bayes factor and the likelihood ratio are identical, and capture the bare essentials of an account of evidence without any appeal to prior probability. The data D constitute E (evidence) for H1&A1& B against H2&A2&B if and only if their likelihood ratio is greater than one. An immediate corollary of the BF(LR) equation is that there is equal evidential support for both hypotheses only when BF(LR)=1. Note that in this equation if $1< BF(LR) \leq 8$, then D is often said to provide weak evidence for H1 against H2, while when $BF(LR) > 8$, D provides strong evidence. This cut-off point is otherwise determined contextually and may vary depending on the nature of the problem. As is well-known, some data may provide very strong evidence for a hypothesis over its negation without confirming it strongly enough to make it more probable than its prior probability. It is also possible that confirmation can be very strong, but the evidence is weak.

We call our account a "robust Bayesian" account. We are subjective Bayesians insofar as, like most Bayesians, we allow and endorse an agent's subjective degree of belief to capture her prior probability, which thus influences the entire posterior probability calculation with regard to the confirmation question. At the same time, the evidential relationship between two competing hypotheses, data, and background assumption is objective since the evidential relationship holds true independent of an agent's subjective probabilities for competing hypotheses. According to our account, the data provide evidence for a hypothesis over its alternative *independent* of what the agent knows or believes. In this sense, we are subjective Bayesians regarding the belief/confirmation question, but objective Bayesians regarding the evidence question. This is one reason why our Bayesianism is unique.

[1] These probabilities of the data are also known as marginal or integrated likelihoods; some authors also denote them as "evidence" (e.g., MacKay 2004; Bailer-Jones 2017), which must not be confused with our account of evidence that always implies a comparison between *two* competing simple statistical hypotheses.

Generalized Account and Prediction

Having addressed the first two questions relating to scientific inference, we will address the third question, i.e., what does the datum tell us about the *predictive accuracy* of the hypothesis? We respond by considering hypotheses with adjustable parameters.[2] In Bayes' theorem, the marginal probability, Pr(D), is also called the predictive probability; it uses data to find the best function to predict a future data point. If one is interested in the predictive accuracy of a hypothesis, then Bayesians suggest that one should use the predictive distribution. In the maximum likelihood estimation (MLE) approach, in contrast, one uses data to find the best value for the parameter and predicts a future data by estimating the parameter. If, however, an investigator is interested in maximizing the predictive accuracy of a model, then she will be better off using the posterior predictive distribution instead of MLE approach crucial to some non-Bayesian approaches (e.g., Forster & Sober 2004) as the former is able to provide a better prediction.

A Decision-Theoretic Account and Acceptance

Finally, we address the fourth question posed: given the datum, what should we *do*? We accept hypotheses because of their ability to make true predictions or explain some phenomenon. Two questions arise in connection with acceptance of a hypothesis: i) *what* is acceptance of a hypothesis and ii) if we sometimes accept a hypothesis, then what *justifies* this acceptance? To answer these questions, we provide a Bayesian theory of acceptance. Building on Bas van Fraassen's theory (van Fraassen 1980), we defend a double aspect theory of acceptance: a) the belief aspect that states my degrees of belief in a hypothesis and b) the pragmatic aspect that states my non-epistemic reasons for pursuing a hypothesis, such as thinking it is important that a patient takes self-responsibility when fighting his or her cancer. In our account, we also have a justification for this double-aspect account of acceptance. A) Like Bayesians, in our view, an agent's degrees of belief must obey the probability calculus and any change in her degrees of belief must be done in accordance with the rule of conditionalization. B) As Bayesians, we justify the agent's pragmatic reasons for pursuing a hypothesis by invoking the principle of maximizing expected utility (hereafter, MEU) calculated on the basis of the probability of that action multiplied by its utility. According to MEU, in a given decision situation, the decision maker should choose the alternative with maximal expected utility. As Bayesians, we contend that in a decision situation one ought to accept the hypothesis which has a higher expected utility than any other. Sometimes, it may happen that we accept a hypothesis that has a lower probability than the other hypotheses in a domain, because it has a significantly higher utility. As a result, based on our expected utility calculation, we end up getting a higher expected utility if we accept the hypothesis. In contrast, we may

[2] These are also referred to as "models". By a "model" we mean a mathematical entity that specifies a probability distribution over a range of data which might be collected. In principle, models need to be distinguished from hypotheses which, unlike models, are statements that do not necessarily contain a quantitative assertion about how probable data are under them, although both terms are sometimes used interchangably if the hypothesis can be formulated as a mathematical relation with adjustable parameters. As a didactic example, an economic model might say that the price of apples is linearly related (with specified intercept, slope and error variance) to the supply of apples, while an economic hypothesis might only state that the price of apples decreases with their supply.

embrace a hypothesis that has a lower utility, but which has an appreciably higher probability. In the end, we embrace it, because we obtain a higher expected utility if we embrace it. We are like subjective Bayesians (engaged in decision theory) in the matter of accepting a hypothesis or not, as the pragmatic dimension of hypothesis acceptance enters into our characterization of subjective Bayesianism.

Unique Features of Our Research Program

There are two distinctive features of this project. First, it is an exercise in "Hybrid Bayesianism." This Bayesian framework is "hybrid" because it contains both subjective and objective elements of scientific theorizing. It is different from subjective Bayesianism because it has a part called "evidence" which is objective. It differs from objective Bayesianism because it contains the significance of belief or confirmation, which is subjective. Currently we are the only hybrid Bayesians around. So, there might be a natural interest among philosophers, statisticians, and methodologists in our novel stance. Second, our research critically questioning the stance of Evidence Based Medicine on ketogenic therapy, or what is called Alternative and Complementary Medicine in general, has been partly motivated by the debate between Karl Popper and Thomas Kuhn on the correct scientific methodology. They hold seemingly opposing accounts of science. Popper emphasizes the objective view of testing in terms of bold conjectures and their subsequent refutations, whereas Kuhn offers a subjective view based on a paradigm-centric hypothesis appraisal in which one paradigm is replaced by another dramatized by Kuhn himself as a "Duck-Rabbit" transformation. According to Kuhn, when an old paradigm is replaced by a new paradigm, what looked like a duck before presently appears to be a rabbit from the perspective of this new paradigm. We feel the need to appreciate these objective and subjective elements in scientific methodology. However, no quantitative work has yet been done to address it. Our research program will serve to fill a gap in the literature while formulating a systematic account in which Popper's and Kuhn's insights are neatly incorporated in a hybrid Bayesian framework.

Appendix B. WinBUGS code for the enthusiastic prior model

We here provide the WinBUGS code of our model using the enthusiastic prior (EP) which builds upon the code provided in Appendix A of Jones et al. (2009).

```
model
        {
        for (i in 1:species){
                for (j in 1:intervention){
                        tau[i,j] <- 1/pow(se.y[i,j],2)
                        y[i,j] ~ dnorm(theta[i,j],tau[i,j])
                        tau[i,j] ~ dgamma(1.0E-3,1.0E-3)

                        mu[i,j] <- alpha[i] + gamma[j]
                        theta[i,j] ~ dnorm(mu[i,j],tau.btw)
                        mr[i,j] <- exp(mu[i,j])
                        }
                }

        alpha[1:5] ~ dmnorm(mu.alpha[],T.alpha[,])
        gamma[1:4] ~ dmnorm(mu.gamma[],T.gamma[,])
        T.alpha[1:5,1:5] ~ dwish(Ralpha[,],5)
        T.gamma[1:4,1:4] ~ dwish(Rgamma[,],4)
        Sigma.alpha[1:5,1:5] <- inverse(T.alpha[ , ])
        Sigma.gamma[1:4,1:4] <- inverse(T.gamma[ , ])

                tau.btw <- 1/sigma2.btw
                sigma2.btw <- pow(sigma.btw,2)
                sigma.btw ~ dnorm(0.5,100)
        }

Data
list(species = 5, intervention = 4,

y = structure(.Data = c(0.3436, 0.2311, NA, 0.5596,
                        -0.0186 , 0.1345, 0.3563, NA,
                        0.2261, 0.5760514, 0.5415,NA,
                        0.6325 ,  NA, 0.3556, NA,
                        0.0117, NA, NA, NA), .Dim = c(5,4)),

se.y = structure(.Data = c(0.2842, 0.19715, 0.000001, 0.1532,
                           0.3529, 0.0305, 0.03761, 0.000001,
                           0.0956, 0.0961, 0.3219, 0.000001,
                           0.5048, 0.000001, 0.05007, 0.000001,
                           0.3228, 0.000001, 0.000001, 0.000001), .Dim = c(5,4)),

Ralpha = structure(.Data=c(0.142884,0,0,0,0,
                           0,0.0064,0.0063,0.0063,0,
                           0, 0.0063,0.0064,0.0063,0,
                           0, 0.0063,0.0063,0.0064,0,
                           0, 0,0,0,1), .Dim=c(5,5)),
Rgamma = structure(.Data=c(1,0,0,0,
```

```
                    0,1,0,0,
                    0,0,1,0,
                    0,0,0,1), .Dim=c(4,4)),

mu.alpha = c(0.336,0.117,0.117,0.117,0),
mu.gamma = c(0,0,0,0))

Inits:chain 1 list(
    T.alpha =  structure(.Data = c(0,0,0,0,0,
                    0,0,0,0,0,
                    0,0,0,0,0,
                    0,0,0,0,0,
                    0,0,0,0,0), .Dim = c(5,5)),
    T.gamma =  structure(.Data = c(0,0,0,0,
                    0,0,0,0,
                    0,0,0,0,
                    0,0,0,0), .Dim = c(4,4)),
    alpha = c(0,0,0,0,0),

    sigma.btw = 0.5

    gamma = c(0,0,0,0),

    theta = structure(.Data = c(0,0,0,0,
                    0,0,0,0,
                    0,0,0,0,
                    0,0,0,0,
                    0,0,0,0), .Dim = c(5,4)),

    y = structure(.Data = c(NA, NA, 0, NA,
              NA, NA, NA, 0,
              NA, NA, NA, 0,
                 NA, 0, NA, 0,
              NA, 0, 0, 0), .Dim = c(5,4)))
```

Appendix C. Supplementary Tables

Prior	$\bar{\sigma}$ (95% CrI)	$\overline{H}(\theta_{i,j}|\bar{\sigma}^2)$	DIC	p_D
Skeptical prior SP1	0.44 (0.24-0.63)	0.57	−2.298	10.928
Skeptical prior SP2	0.41 (0.22-0.60)	0.52	−3.099	10.476
Fundamentalist skeptical FSP	0.42 (0.23-0.62)	0.55	−2.758	10.635
Relational priors RP1	0.40 (0.21-0.60)	0.51	−3.085	10.426
Relational priors RP2	0.40 (0.21-0.60)	0.50	−3.265	10.328
Relational priors RP3	0.40 (0.21-0.60)	0.50	−3.265	10.327
Mechanistic prior MP1	0.41 (0.22-0.60)	0.52	−3.119	10.471
Mechanistic prior MP2	0.39 (0.21-0.59)	0.49	−3.714	10.168
Enthusiastic prior EP	0.386 (0.205-0.582)	0.47	−3.634	10.086
Enthusiastic prior EP+MP1	0.386 (0.205-0.580)	0.47	−3.648	10.086
Enthusiastic prior EP+MP2	0.368 (0.193-0.566)	0.42	−4.156	9.778

Table S1: Posterior estimates for σ and differential entropy $\overline{H}(\theta_{i,j}|\bar{\sigma}^2)$ in relation to the different model priors, where $\bar{\sigma}$ denotes the posterior median. DIC values and effective number of parameters p_D are also given.

mean	variance	KD	KD+	CR	CR+
0.25	0.1	1.22 (0.80-1.89)	1.40 (0.95-2.07)	1.63 (0.96-2.52)	1.68 (1.06-2.54)
0.25	0.01	1.24 (0.76-2.00)	1.39 (0.91-2.13)	1.58 (0.91-2.63)	1.65 (1.00-2.66)
0.25	0.005	1.25 (0.78-2.03)	1.39 (0.90-2.16)	1.56 (0.89-2.66)	1.64 (0.99-2.69)
0.5	0.1	1.22 (0.78-2.00)	1.40 (0.91-2.15)	1.61 (0.91-2.61)	1.66 (0.99-2.63)
**0.5**	**0.01**	**1.29 (0.68-2.45)**	**1.39 (0.73-2.59)**	**1.50 (0.70-3.21)**	**1.54 (0.73-3.29)**
0.5	0.005	1.30 (0.66-2.58)	1.38 (0.70-2.70)	1.50 (0.66-3.33)	1.52 (0.70-3.42)
1	0.1	1.25 (0.70-2.36)	1.39 (0.75-2.49)	1.56 (0.73-3.05)	1.61 (0.76-3.10)
1	0.01	1.28 (0.43-3.75)	1.32 (0.44-4.03)	1.40 (0.40-4.97)	1.35 (0.39-4.98)
1	0.005	1.28 (0.42-3.83)	1.31 (0.43-4.16)	1.39 (0.38-5.14)	1.34 (0.38-5.17)

Table S2: Posterior estimates for the MR depending on different choices of the prior for σ, while the priors for α and γ were fixed at SP2 in all cases. The bold numbers are those used in the main analysis.

	KD	KD+	CR	CR+
**Original study estimates**	**1.41 (0.61-2.21)**	**1.26 (0.77-1.75)**	**NA**	**1.75 (1.22-2.28)**
Skeptical prior SP1	1.32 (0.59-2.89)	1.37 (0.63-2.95)	1.51 (0.58-3.87)	1.69 (0.68-4.16)
Skeptical prior SP2	1.32 (0.68-2.53)	1.37 (0.72-2.60)	1.53 (0.71-3.21)	1.55 (0.73-3.30)
Fundamentalist skeptical FSP	1.31 (0.65-2.58)	1.34 (0.68-2.65)	1.51 (0.67-3.35)	1.47 (0.63-3.33)
Relational priors RP1	1.35 (0.63-2.86)	1.39 (0.71-2.78)	1.53 (0.64-3.59)	1.65 (0.75-3.64)
Relational priors RP2	1.34 (0.63-2.83)	1.40 (0.71-2.77)	1.54 (0.65-3.60)	1.65 (0.75-3.65)
Relational priors RP3	1.34 (0.62-2.82)	1.40 (0.71-2.79)	1.54 (0.65-3.58)	1.65 (0.75-3.66)
Mechanistic prior MP1	1.26 (0.64-2.39)	1.38 (0.73-2.64)	1.45 (0.67-3.03)	1.67 (0.78-3.60)
Mechanistic prior MP2	1.40 (0.75-2.52)	1.46 (0.86-2.50)	1.43 (0.75-2.60)	1.49 (0.86-2.60)
Enthusiastic prior EP	1.44 (0.80-2.55)	1.51 (0.85-2.59)	1.75 (0.92-3.21)	1.59 (0.79-3.28)
Enthusiastic prior EP+MP1	1.39 (0.76-2.49)	1.52 (0.85-2.67)	1.68 (0.87-3.08)	1.74 (0.85-3.61)
Enthusiastic prior EP+MP2	1.57 (0.92-2.57)	1.56 (0.95-2.52)	1.61 (0.92-2.66)	1.58 (0.95-2.63)

Table S3: Posterior estimates of the MR for humans with three replicate animal studies excluded. The numbers give the median and 95% credible interval obtained with different prior distributions.

	KD	KD+	CR	CR+
**Original study estimates**	**NA**	**1.26 (0.77-1.75)**	**NA**	**1.75 (1.22-2.28)**
Skeptical prior SP1	1.11 (0.35-3.74)	1.30 (0.52-3.19)	1.35 (0.41-4.39)	1.69 (0.66-4.26)
Skeptical prior SP2	1.19 (0.51-2.85)	1.34 (0.65-2.69)	1.42 (0.59-3.39)	1.53 (0.69-3.21)
Fundamentalist skeptical FSP	1.17 (0.46-3.03)	1.30 (0.60-2.77)	1.40 (0.53-3.58)	1.45 (0.62-3.26)
Relational priors RP1	1.21 (0.40-3.68)	1.34 (0.60-2.98)	1.39 (0.47-4.06)	1.63 (0.73-3.62)
Relational priors RP2	1.19 (0.39-3.66)	1.34 (0.60-3.02)	1.38 (0.48-4.09)	1.64 (0.73-3.62)
Relational priors RP3	1.19 (0.40-3.63)	1.34 (0.60-3.00)	1.38 (0.48-4.10)	1.64 (0.73-3.61)
Mechanistic prior MP1	1.11 (0.48-2.63)	1.32 (0.64-2.69)	1.31 (0.55-3.13)	1.64 (0.74-3.53)
Mechanistic prior MP2	1.30 (0.59-2.84)	1.46 (0.80-2.58)	1.33 (0.60-2.91)	1.48 (0.83-2.68)
Enthusiastic prior EP	1.45 (0.69-2.90)	1.50 (0.81-2.70)	1.66 (0.82-3.26)	1.60 (0.77-3.25)
Enthusiastic prior EP+MP1	1.37 (0.64-2.74)	1.50 (0.80-2.72)	1.58 (0.77-3.11)	1.73 (0.83-3.60)
Enthusiastic prior EP+MP2	1.55 (0.82-2.86)	1.57 (0.95-2.59)	1.58 (0.84-2.92)	1.59 (0.94-2.67)

Table S4: Same as Table 3 in the main manuscript, but without using the datum from Rieger et al. (2014).

References

Bailer-Jones CAL. 2017. Practical Bayesian Inference. A Primer for Physical Scientists. Cambridge, United Kingdom: Cambridge University Press.

Forster M, Sober E. 2004. Why likelihood? In: Taper ML, Lele SR, editors. Nat Sci Evid. 1st ed. Chicago & London: The University of Chicago Press; p. 153–190 (with discussion).

van Fraassen BC. 1980. The Scientific Image. 1st ed. Oxford: Oxford University Press.

Jones DR, Peters JL, Rushton L, Sutton AJ, Abrams KR. 2009. Interspecies extrapolation in environmental exposure standard setting: A Bayesian synthesis approach. Regul Toxicol Pharmacol. 53:217–225.

Kass RE, Raftery AE. 1995. Bayes Factors. J Am Stat Assoc. 90:773–795.

MacKay DJC. 2004. Information Theory, Inference, and Learning Algorithms. 3rd ed. Cambridge, United Kingdom: Cambridge University Press.

Rieger J, Bähr O, Maurer GD, Hattingen E, Franz K, Brucker D, Walenta S, Kämmerer U, Coy JF, Weller M, Steinbach JP. 2014. ERGO: A pilot study of ketogenic diet in recurrent glioblastoma. Int J Oncol. 44:1843–1852.

Royall R. 1997. Statistical Evidence: A Likelihood Paradigm. New York: Chapman and Hall/CRC.

Royall R. 2004. The likelihood paradigm for statistical evidence. In: Taper ML, Lele SR, editors. Nat Sci Evid. 1st ed. Chicago & London: The University of Chicago Press; p. 119–152 (with discussion).
